# Associations between the spread of COVID-19 and end-of-life circumstances in the non-infected population of Sweden

**DOI:** 10.1177/14034948231216197

**Published:** 2023-12-28

**Authors:** Stefan Sennfält, Christel Hedman, Carl Johan Fürst

**Affiliations:** 1 Department of Neurology, Karolinska University Hospital, Sweden; 2 Department of Clinical Neuroscience, Karolinska Institutet, Sweden; 3 Department of Clinical Sciences, Lund University, Sweden; 4 Institute for Palliative Care, Lund University and Region Skåne, Sweden; 5 Department of Molecular Medicine and Surgery, Karolinska Institutet, Sweden; 6 R&D Department, Stockholms Sjukhem Foundation, Sweden

**Keywords:** COVID-19, Palliative care, Death, Loneliness

## Abstract

**Aims:**

Since its outbreak in 2020, the COVID-19 pandemic has directly caused the premature death of millions. However, indirect consequences, such as social restrictions, have affected a far greater number. We explored the association between the spread of COVID-19 and end-of-life circumstances in the infected and non-infected population in Sweden.

**Methods:**

In this descriptive, population-based, observational study, we primarily used data from the Swedish National Registry of Palliative Care, which covers about 60% of all deaths in Sweden. We explored the association between the spread of COVID-19 and place of death, people present at death and end-of-life symptoms using regression analyses.

**Results:**

The study included 190,291 individuals who died in any region of Sweden from 1 January 2019 to 30 June 2022, of which 10,646 were COVID-19 cases. Correlated to the temporal and geographical spread of COVID-19, there was a greater proportion of individuals dying without the presence of their next-of-kin, and consequently more people dying alone, both in those with and without COVID-19. There was a similar pattern of a greater proportion of deaths taking place in nursing homes and in the individual’s own home. However, we did not find substantial associations to reported symptoms, such as anxiety or confusion.

**Conclusions:**

**This study shows the profound effects of the COVID-19 pandemic on end-of-life circumstances in both the infected and non-infected population in Sweden. As we prepare for future pandemics, there is a need to develop strategies to minimise the impact on non-infected individuals.**

## Introduction

The COVID-19 pandemic has had devastating effects since its outbreak in 2020. The number of deaths directly caused by the disease in 2020 and 2021 is estimated to be almost 20 million worldwide [1], with the disease disproportionately affecting the elderly and frail [2]. Many authorities imposed measures of varying severity in an effort to limit the spread of the disease, including travel restrictions, complete or partial lockdowns, and social distancing [3]. These measures had a significant impact on the quantitative and qualitative output of healthcare services. There are numerous reports of substantial challenges to these services in the COVID-19 era [4,5], many describing a lower standard of care due to strain and restrictions [6,7]. However, the indirect consequences of the pandemic are much more difficult to quantify and have affected a far greater number of individuals. Social interactions were restricted within healthcare (e.g. a decrease in in-person healthcare contacts [8,9] and restrictions in visiting policies [10,11]) and in society as a whole. Many studies report profound negative effects of social distancing on psychological well-being, particularly among the elderly [7,12–14] and likely more severely effecting those dependent on close care and social contact, such as individuals with palliative care needs.

Sweden adopted a distinct strategy implementing relatively lenient mandatory closures and placing greater emphasis on voluntary restrictions [15,16]. The primary focus was on curbing the transmission of the virus through measures such as maintaining physical distance, limiting public gatherings and implementing strict hygiene practices. The extent of the restrictions varied over time, primarily influenced by the level of viral spread. Initially, testing was conducted conservatively, primarily targeting inpatients and healthcare workers due to the limited availability of testing equipment. However, testing had become more widely accessible to the general public in various regions of the country by the summer of 2020.

The Swedish healthcare system operates under a universal framework and the responsibility for end-of-life care is shared between regions and municipalities [17]. Hospitals, including specialised palliative care, fall under the jurisdiction of the regions, whereas home-based care and nursing homes are managed by the municipalities. As a result, there is some geographical variation influenced by local financial and political circumstances. In recent years, institutional changes have contributed to a decrease in the proportion of people dying in hospital. As of 2017, an estimated 39% died in hospital, 36% in assisted living facilities (including palliative care units) and 19% in their own homes [18].

Assessing the outcomes of implemented measures is essential for developing optimal strategies for future pandemics. This evaluation should go beyond assessing the impact solely on infected individuals. However, there is currently a lack of systematic research investigating the effects of the COVID-19 pandemic on end-of-life circumstances within the general population. This descriptive, population-based, observational study aims to address this gap by examining end-of-life circumstances in Sweden, including infected and non-infected individuals, during the COVID-19 era (2020–2022). The study uses data from the Swedish National Registry of Palliative Care (SNRPC) to explore various key aspects, including place of death, people present at death and end-of-life symptoms.

## Methods

### Study population

The study included 190,291 individuals registered in the SNRPC who died in any region of Sweden from 1 January 2019 to 30 June 2022, regardless of the cause of death.

### Data

Data on age, sex, place of death, people present at death and symptoms at the end of life were obtained from the SNRPC, which has contributed to research and the development of palliative care in Sweden since 2006 [19]. The registry is based on a validated 30-item end-of-life questionnaire that covers important aspects of quality of care during the last week of life. The questionnaire is completed online by a staff member, typically a nurse or physician, providing care to the individual at the time of their passing. This process takes place in various healthcare settings, including hospitals, nursing homes, palliative care units and primary care facilities. The full questionnaire is mainly completed if the death was anticipated and the registry was estimated to cover about 60% of all deaths in Sweden in 2020 [18].

For most variables, the amount of missing data was small: in two individuals (0.0%) for ‘sex’, in 4187 (2.2%) for ‘people present at death’ and no missing data for ‘place of death’. However, for ‘symptoms at the end of life’ there was a substantial proportion of missing data: in 23,665 individuals (12.4%) for pain, in 32,322 (17.0%) for anxiety, in 38,973 (20.5%) for confusion, in 35,325 (18.6%) for nausea and in 26,054 (13.7%) for dyspnoea.

National and regional data on the total number of inhabitants and deaths in Sweden were obtained from Statistics Sweden with an almost complete coverage [20]. National and regional data on COVID-19-related deaths were obtained from the Swedish Public Health Agency (SPHA) and were used as an indicator of the temporal and geographical spread of the disease. These data were related to the number of inhabitants in the different regions of Sweden in December 2020 to yield the number of cases per 100,000 inhabitants.

### Measures and definitions

#### COVID-19 cases

The SNRPC registers COVID-19 status as suspected or confirmed, both of which are referred to as COVID-19 cases in this paper.

#### First, second and third waves of the COVID-19 pandemic

There were three distinct waves of COVID-19 in Sweden during the study period, each lasting for about six months: the first wave from March to August 2020; the second wave from October 2020 to March 2021; and the third wave from December 2021 to May 2022.

#### People present at death

Defined as those present when the individual died: next-of-kin (with or without healthcare staff present); only healthcare staff; or nobody (dying alone).

#### Place of death

Defined as the location where the individual died: their own home; palliative care unit; nursing home; short-term assisted living; hospital; or other.

#### Symptoms at the end of life

Defined as the symptoms reported in the week preceding death by the healthcare professionals caring for the individual. In the SNRPC, these are specified as ‘completely relieved’, ‘partially relieved’ or ‘not at all relieved’. In this study, these categories were dichotomised into ‘present’ (completely relieved, partially relieved or not at all relieved) or ‘not present’. We included symptoms considered to reflect general well-being at the end of life (pain, anxiety, nausea and confusion), but we also included dyspnoea, which is more likely to be specifically associated with COVID-19 infection.

#### Temporal and geographical spread of COVID-19

This was indicated by the number of COVID-19-related deaths as reported by the SPHA.

### Statistical analyses

Categorical variables were summarised as proportions (percent) and the χ^2^ test was performed to assess for differences between groups. The continuous variable ‘age at death’ was reported as a median with the interquartile range (IQR) and Student’s *t*-test was used to assess for differences between groups.

Group-level linear regression analyses were conducted to examine the relationship between the number of COVID-19-related deaths per month (predictor, independent variable) and the proportional distribution of variables such as ‘place of death,’ ‘people present at death’ and ‘symptoms at the end of life’ during the same month (outcome, dependent variable). Separate models were created for each outcome – for example, for the variable ‘people present at death’: nobody; next-of-kin; or with healthcare staff only. Linear regression models were also created to explore the relationship between the geographical spread of COVID-19 and the proportion of individuals dying alone. The number of COVID-19-related deaths during the first wave of the pandemic (March–August 2020) in each separate region was used as the predictor, independent variable and the proportion of individuals dying alone was used as the outcome, dependent variable. All regression analyses were performed both including and excluding COVID-19 cases and the result were reported as the standardised beta (Std. β), rounded to two decimals. For all tests, the level of significance was set to *p*<0.05 if not indicated otherwise. All statistical analyses were conducted in IBM SPSS Statistics version 28.

## Results

### Subject characteristics and number of deaths

The study included 190,291 individuals (46.7% men) with a median age at death of 84 years (IQR 14 years) registered in the SNRPC (Table I). The total number of COVID-19 cases was 10,646 (5.6%): 10.9% for 2020; 5.5% for 2021; and 5.1% for 2022. These individuals were more often men (53.1%) and slightly older (median age 85 years, IQR 12 years). The total number of deaths during the same period (as reported by Statistics Sweden) was 325,178, of which 19,157 (5.9%) were COVID-19-related deaths (as reported by the SPHA). Thus, there was an estimated 58.9% (190,291/325,178) coverage of the SNRPC to a similar proportion of COVID-19 cases compared with the Statistics Sweden/SPHA data.

**Table I.** Subject characteristics and end-of-life circumstances in infected and non-infected individuals.

	Total (*N*=190,291)	Non-COVID-19 cases (*n*=179,645) (94.4%)	COVID-19 cases (*n*=10,646) (5.6%)
**Subject characteristics**			
Sex (male)^a^	88,961 (46.7)	83,308 (46.4)	5653 (53.1)
Median (IQR) age at death (years)^a^	84 (14)	84 (14)	85 (12)
**Place of death** ^a^			
Own home	26,242 (13.8)	25,902 (14,4)	340 (3.2)
Palliative care unit	24,075 (12.7)	23,699 (13,2)	376 (3.5)
Nursing home	70,235 (36.9)	66,242 (36,9)	3993 (37.5)
Short-term assisted living	13,533 (7.1)	1121 (7.3)	412 (3.9)
Hospital ward	54,887 (28.8)	49,430 (27.5)	5457 (51.3)
Other location	1319 (0.7)	1251 (0.7)	68 (0.6)
**People present at death** ^a^			
Nobody	36,980 (19.9)	33,657 (19.1)	3323 (32.6)
Next-of-kin	91,770 (49.3)	88,994 (50.6)	2776 (27.2)
Healthcare staff only	57,354 (30.8)	53,257 (30.3)	4097 (40.2)
**Symptoms at the end of life** ^a^			
Pain	120,776 (72.5)	115,098 (72.9)	5678 (65.3)
Anxiety	91,829 (58.1)	86,577 (57.9)	5252 (63.0)
Confusion	39,290 (26.0)	36,842 (25.6)	2448 (31.9)
Dyspnoea	44,301 (27.0)	39,891 (25.7)	4410 (50.3)
Nausea	22,313 (14.4)	21,631 (14.7)	682 (8.9)

IQR: interquartile range. Data presented as *n* (%).

a Significant difference between individuals with and without COVID-19, *p*<0.01.

During the first, second and third waves of the COVID-19 pandemic, there were substantial increases in the number of deaths, mostly accounted for by COVID-19 cases (Supplementary Figure 1, available online). For instance, from February to April 2020 (first wave), there was an increase in the total number of deaths registered in the SNRPC from 4633 to 6554 (difference 1921), of which 1556 were COVID-19 cases. The temporal trend in the number of deaths (people with and without COVID-19) registered in the SNRPC mirrored the numbers reported by Statistics Sweden and the SPHA.

### Place of death

**Figure 1.**
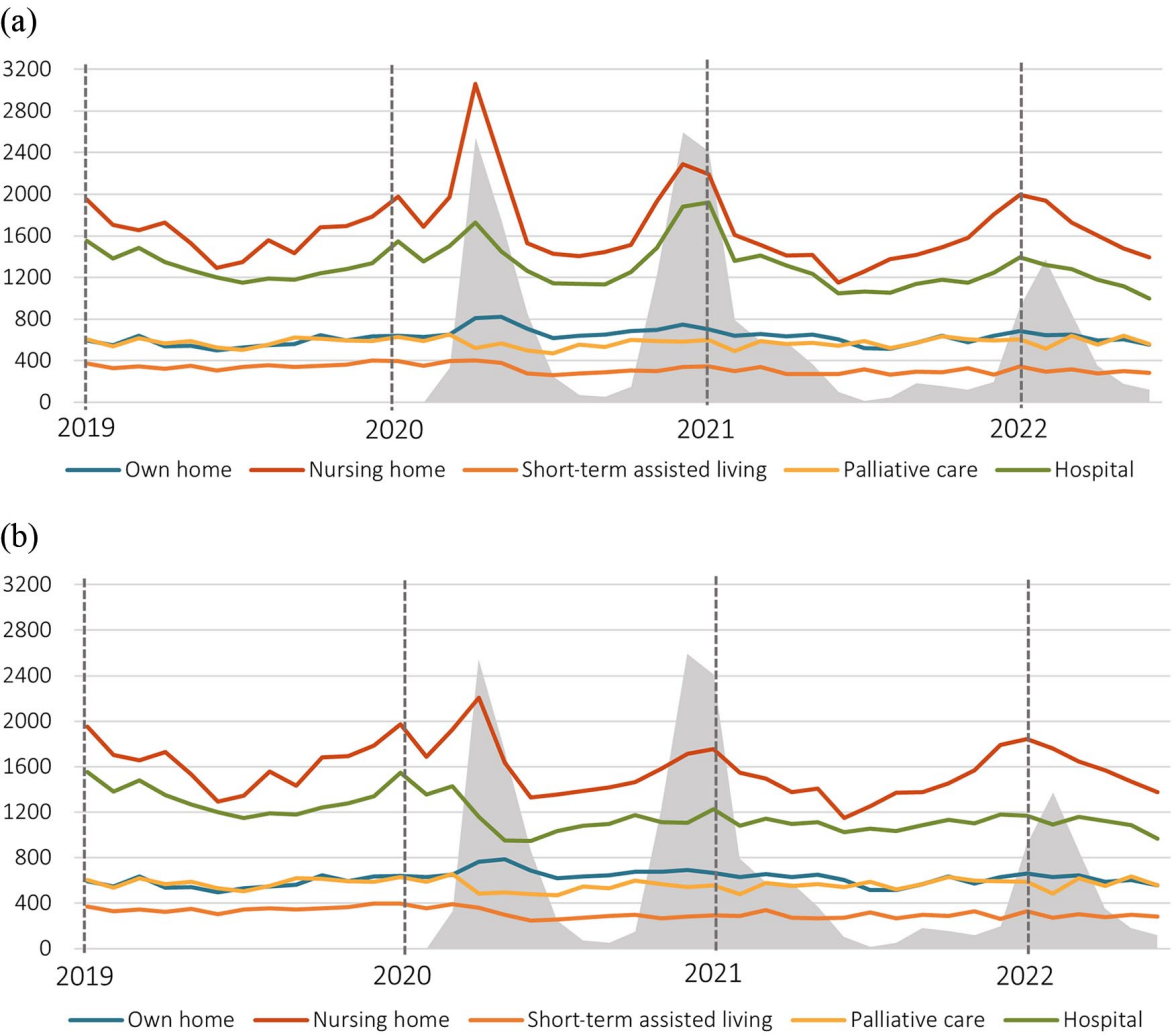
**Absolute number of deaths at various locations.** Shown per month for (a) all deaths (*n*=190,291) and (b) excluding COVID-19 cases (*n*=179,645). The category ‘other locations’ (*n*=1319; 0.7%) is not shown. The area in grey represents the spread of COVID-19 as indicated by the number of COVID-19-related deaths.

During the first, second and third waves of the COVID-19 pandemic, there was a significant increase in the absolute number and proportion of deaths at nursing homes and in the individual’s own home, which, although attenuated, persisted after excluding COVID-19 cases (Figure 1(b), Table II). Also, the number of deaths in hospital increased during all three waves of the pandemic. However, when excluding COVID-19 cases, the increase in hospital deaths did not persist, but there was instead a significant proportional decrease. The absolute number of deaths at palliative care units varied little, although there was a significant proportional decrease.

**Table II.** Linear regression analyses of the association between the spread of COVID-19 and variables reflecting end-of-life circumstances. Separate models were constructed for each outcome of interest. The number of COVID-19-related deaths per month was used as the predictor variable and the proportional distribution in each outcome was used as the dependent variable. The place of death category ‘other locations’ (*n*=1319; 0.7%) is not shown.

	All cases (*N*=190,291)		Non-COVID-19 cases (*n*=179,645)	
	Std. β	*p*	Std. β	*p*
**Place of death**				
Own home	−0.13	0.405	0.45	0.003
Palliative care unit	−0.75	<0.001	−0.52	<0.001
Nursing home	0.62	<0.001	0.62	<0.001
Short-term assisted living	−0.64	<0.001	−0.42	0.05
Hospital ward	0.18	0.263	−0.69	<0.001
**People present at death**				
Nobody	0.85	<0.001	0.66	<0.001
Next-of-kin	−0.90	<0.001	−0.80	<0.001
Healthcare staff only	0.85	<0.001	0.80	<0.001
**Symptoms at the end of life**				
Pain	−0.57	<0.001	−0.47	0.002
Anxiety	−0.11	0.507	−0.28	0.070
Confusion	0.08	0.619	−0.16	0.305
Nausea	−0.65	<0.001	−0.43	0.004
Dyspnoea	0.81	<0.001	−0.16	0.328

### People present at death

**Figure 2.**
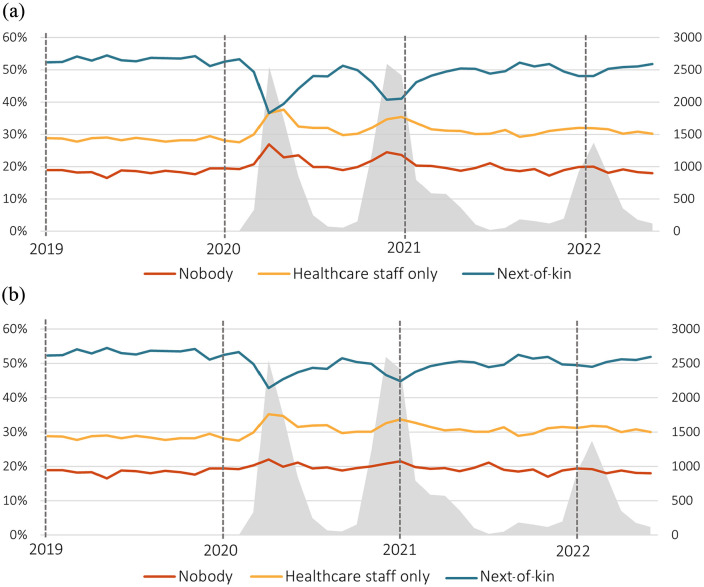
People present at death. Shown per month for (a) all deaths (*n*=186,104) and (b) excluding COVID-19 cases (*n*=175, 908). The three different proportions sum to 100%. The area in grey represents the spread of COVID-19 as indicated by the number of COVID-19-related deaths on the secondary axis to the right.

During all three waves of the pandemic, there was a significant decrease in the proportion of deaths where next-of-kin were present (Std. β −0.90) and, despite the presence of healthcare staff in many of these instances, there was a significant increase in the proportion of individuals dying alone (Std. β 0.85) (Figure 2(a), Table II). When excluding COVID-19 cases, these changes were less prominent, although still significant (Std. β −0.80 and 0.66, respectively) (Figure 2(b), Table II).

### Geographical distribution of individuals dying alone and association to the spread of COVID-19

**Figure 3.**
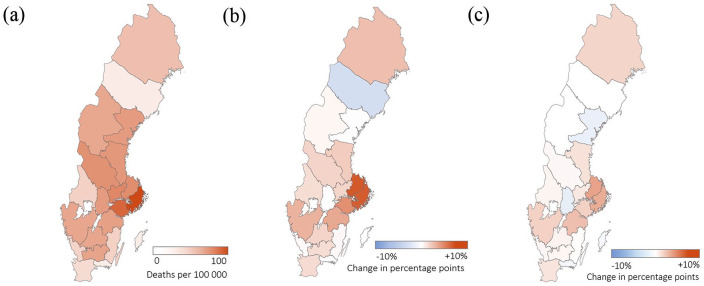
Association between the proportion of individuals dying alone and the geographical spread of COVID-19. (a) Geographical spread of COVID-19 during the first wave of the pandemic (March–August 2020) and the change in the proportion of individuals dying alone compared to the year 2019 (percentage points) for (b) all deaths and (c) excluding COVID-19 cases. For the first wave and for the year 2019, the total number of deaths were 28,767 and 52,394, respectively.

To further verify the effects of the spread of COVID-19 on end-of-life circumstances, we explored the association between regional spread and the proportion of individuals dying alone. For the first wave of the COVID-19 pandemic (March–August 2020), these were shown to be significantly associated, both when including and excluding COVID-19 cases (Std. β 0.60 and 0.47, respectively). Figure 3 shows the change in the proportion of individuals dying alone for each region compared with the year 2019. However, there was no significant association for the subsequent waves.

### Symptoms at the end of life

During all three waves, there was a significant positive association between the spread of COVID-19 and the prevalence of dyspnoea (Std. β 0.81) that did not, however, persist after excluding COVID-19 cases (Table II). Conversely, there was a significant negative association with pain (Std. β −0.57) and nausea (Std. β −0.65) that did persist after excluding COVID-19 cases (Std. β −0.47 and −0.43, respectively). There was no significant association with reported symptoms of anxiety or confusion, neither when including nor excluding COVID-19 cases.

## Discussion

### Main findings

Our results show the profound effects of the COVID-19 pandemic on both the infected and non-infected population in Sweden, with serious consequences for those in greatest need of support: individuals at the end of life. During all three waves of the pandemic, there was a decrease in the presence of next-of-kin at death and, consequently, a greater proportion of individuals dying alone. A geographical analysis revealed a pattern of larger proportions of individuals dying alone in regions where COVID-19 was more prevalent, providing further evidence of a true association. Also, there was a disproportionate increase in the number of deaths taking place in nursing homes and in individuals’ own homes.

### The findings in context

A greater proportion of individuals dying alone during the COVID-19 pandemic has also been reported by others [6,21] and might be a result of measures adopted in response to viral spread, such as visiting restrictions at healthcare facilities and self-imposed social restrictions. Every individual’s dying experience is unique and numerous factors come into play that influence the likelihood of a ‘good death’ [22] . A central notion in palliative care is the concept of ‘total pain’: to reduce not only physical, but also psychological, social and spiritual suffering [23]. Specifically, human contact is often cited as an essential component of a ‘good death’ [24,25], which makes our results concerning. However, even though we did not specifically measure psychological well-being, we did not find any clear association between the spread of COVID-19 and reported symptoms of anxiety or confusion.

As for the increase in the number of deaths taking place in permanent places of living (nursing homes and individuals’ own homes), this might have several explanations – for instance, increased strain on the healthcare system and a reluctance to send a seriously ill person to hospital or a palliative care unit because of restrictions and fear of contracting COVID-19. Whether the change in the place of death resulted in increased or decreased well-being remains unclear. There is ample evidence that dying at home is often preferred if given the choice [26] and, during the COVID-19 pandemic, individuals might have chosen not to be transferred to an acute care facility or palliative care unit because of fear of not being able to spend time with their next-of-kin. However, although dying in an institution, particularly hospital, is commonly associated with decreased well-being [27,28], the circumstances during the COVID-19 pandemic were unique, affecting care everywhere in an unpredictable manner.

Importantly, the impact of the pandemic on end-of-life care would be expected to differ according to the way in which the healthcare system and palliative care are organised in each setting. Sweden has a universal healthcare system, but decision-making and management is largely decentralised to the regional and municipal levels. Consequently, the measures implemented during the pandemic were likely to differ based on local factors, such as viral transmission. This might have contributed to the observed geographical differences in the proportion of individuals dying alone. Furthermore, the impact of the measures on the general population was likely to have been influenced by the discourse within local society and the narrative presented by local media, which is difficult to analyse retrospectively. Nevertheless, the geographical variation provides further support for a genuine association between viral spread and end-of-life circumstances.

Compared to other countries, Sweden took a unique approach characterised by relatively mild mandatory closures, relying instead on voluntary restrictions [15,16]. Thus, although we can show serious consequences in the Swedish population, there is reason to believe that the effects were greater in countries where measures and restrictions were more severe. This remains to be elucidated.

### Strengths and weaknesses

This is, to the best of our knowledge, the largest study to explore the effects of the COVID-19 pandemic on end-of-life circumstances in the infected and non-infected population. However, the SNRPC only covers about 60% of all deaths in Sweden, primarily those that are anticipated, which affects the generalisability of the findings. For instance, we found a lower proportion of deaths in hospitals compared with registries with a higher coverage: 28.8% in our study compared to 36% (in 2017, as reported by the Swedish Board of Health and Welfare [18]). Also, certain healthcare units (such as those specialising in palliative care) might be more motivated to monitor and report end-of-life care quality data, resulting in selection bias. Specifically, some cases of COVID-19 might not have been reported in the SNRPC, particularly early in the pandemic when testing was not as widespread, resulting in misclassification and skewed results. However, the total number of individuals with COVID-19 in the SNRPC was 10,646, 5.6% of the total number of deaths in this registry, whereas the total number of COVID-19-related deaths reported by the SPHA (which is expected to have a higher coverage) was 19,157, 5.9% of the total number of deaths reported by Statistics Sweden. The small difference might suggest that COVID-19 deaths were, in fact, not disproportionately missed. Although we cannot assert that our findings are representative of the entire Swedish population, our study provides evidence of substantial effects in a sizable portion (about 60%), primarily comprised of anticipated deaths. Moreover, in these cases, the impact of restrictions might be more noticeable because individuals in this group typically receive care over a longer period than those with a sudden or unforeseen death.

## Conclusions

Our results show the profound effects of the COVID-19 pandemic on end-of-life circumstances in infected and non-infected individuals in Sweden. As we prepare for future pandemics, we should take care to develop strategies to minimise the negative effects on the quality of care of all of those not infected. This is particularly important for those in greatest most need of care and human contact, such as individuals at the end of life.

## Supplemental Material

sj-docx-1-sjp-10.1177_14034948231216197 – Supplemental material for Associations between the spread of COVID-19 and end-of-life circumstances in the non-infected population of Sweden Supplemental material, sj-docx-1-sjp-10.1177_14034948231216197 for Associations between the spread of COVID-19 and end-of-life circumstances in the non-infected population of Sweden by STEFAN SENNFÄLT, CHRISTEL HEDMAN and CARL JOHAN FÜRST in Scandinavian Journal of Public Health
