# Supplementary material for: Associations between the spread of COVID-19 and end-of-life circumstances in the non-infected population of Sweden
Source: Scand J Public Health. 2023 Dec 28;52(3):290–8. doi: 10.1177/14034948231216197 (PMC11067408; doi:10.1177/14034948231216197)
Supplement: sj-docx-1-sjp-10.1177_14034948231216197 – Supplemental material for Associations between the spread of COVID-19 and end-of-life circumstances in the non-infected population of Sweden [file sj-docx-1-sjp-10.1177_14034948231216197.docx]

**Supplementary figure**

*Associations between the spread of COVID-19 and end-of-life circumstances in the Swedish non-infected population*


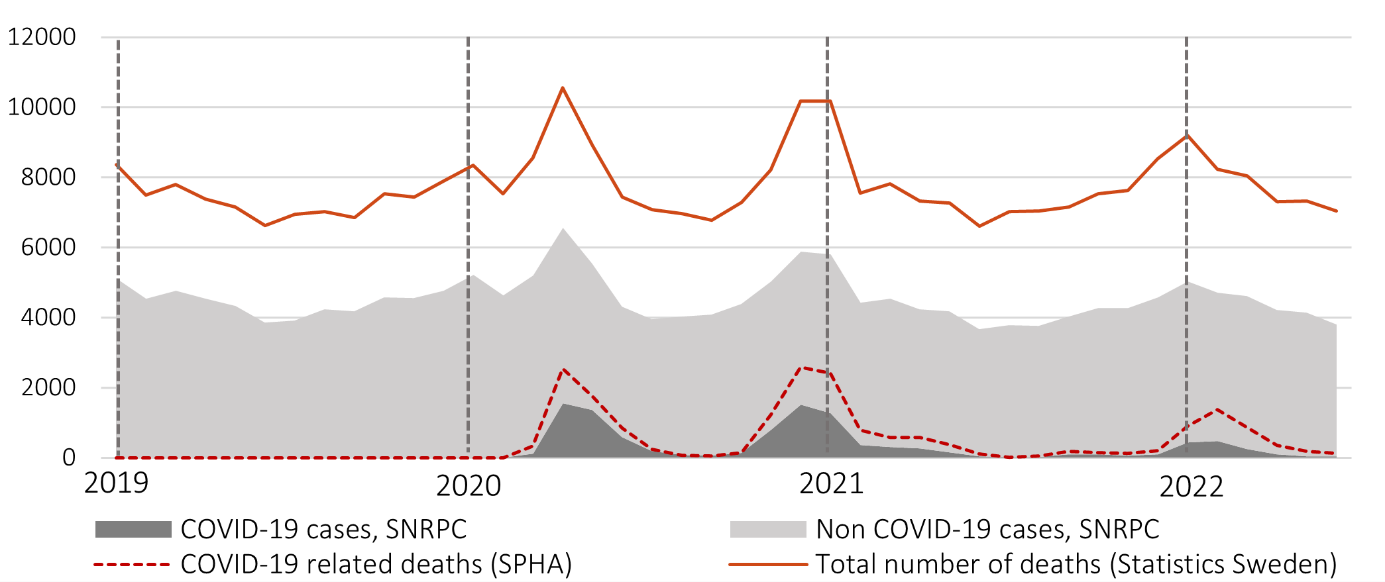


**Supplementary Fig. 1 Deaths in Sweden between January 2019 and June 2022.** Showing the total number of deaths and the proportion of COVID-19 cases registered in the Swedish National Registry of Palliative Care (SNRPC) (light and dark grey areas respectively). The total number of Swedish deaths as reported by Statistics Sweden is and the number of COVID-19 associated deaths as reported by the Swedish Public Health Care Agency (SPHAS) is indicated by the full and dashed red lines, respectively.
